# The role of pharmaceutical nanotechnology in the time of COVID-19 pandemic

**DOI:** 10.2217/fmb-2020-0118

**Published:** 2020-11-20

**Authors:** Lígia N de M Ribeiro, Belchiolina B Fonseca

**Affiliations:** ^1^School of Veterinary Medicine, Federal University of Uberlandia, Uberlândia, Brazil; ^2^Institute of Biotechnology, Federal University of Uberlandia, Uberlândia, Brazil.

**Keywords:** COVID-19, drug toxicity, nanostructured delivery systems, nanotechnology

## Abstract

There is no effective therapy against COVID-19 available so far. In the last months, different drugs have been tested as potential treatments for COVID-19, exhibiting high toxicity and low efficacy. Therefore, nanotechnology can be applied to improve the therapeutic action and minimize the toxicity of loaded drugs. In this review, we summarized the drugs tested as COVID-19 treatment and the advantages of antiviral nanostructured drug-delivery systems. Such systems have demonstrated low *in vitro* toxicity with better *in vitro* antiviral activity than free drugs. We believe that this approach should inspire novel nanostructured drug-delivery systems developments to find efficient COVID-19 treatments. Here, we discuss the remaining challenges for such promising nanosystems to be approved for clinical use.

In 2020, a dramatic global situation related to the viral infection induced by SARS-CoV-2, which causes COVID-19, has been noticed [1]. The first data was recorded in Wuhan, China, December 31^st^ 2019. It has widely spread in China in a few months, and, then, worldwide [2]. This coronavirus-based infection causes various symptoms, even pneumonia and deaths by 7% of infected patients, leading to changes in human habits resulting in the economy and health global crisis [3]. This scenario is continuously changing, once there is no vaccine or effective treatment against COVID-19 available until now.

Experts have moved efforts to provide effective and safe vaccines, treatments and diagnoses tools to contribute to the end of COVID-19 pandemic [4]. Some drugs have become popular concerning the treatment candidates, such as chloroquine or hydroxychloroquine experimental therapies [5]. Other antiviral and antibiotics have been tested against COVID-19 (Table 1). Unfortunately, low bioavailability and high toxicity have been evident in the proposed treatments. Besides, there are only a few reports of *in vivo* efficacy and toxicity tests and clinical trials of such candidates [6]. Pharmacological therapy for COVID-19 is still a challenge.

**Table 1.** Main published works in 2020 regarding drug candidates tested against SARS-CoV, MERS-CoV and SARS-CoV-2, in terms of drug, target binding, type of assays and its traditional uses.

Drug	Target	Assays	Usual treatment	Ref.
Arbidol	Binds to hemagglutinin	*In vitro*	Influenza, arboviruses	[47]
Atazanavir	Inhibition of the 3CL^PRO^	*In silico*,* in vitro*	HIV	[48]
Chloroquine	Immunomodulatory effects, increase endosomal pH required for virus	*In vitro*, clinical trial	Malaria	[49,50]
Dactinomycin^†^	RNA synthesis inhibitor	*In silico*	Cancer	[51]
Darunavir	Protease inhibitor	*In silico*	HIV	[48,52]
Dolutegravir	Protease inhibitor	*In silico*	HIV	[42]
Efavirenz	Inhibition of the 3CL^PRO^	*In silico*	HIV	[42]
Emetine	Inhibits the replication of RNA viruses	*In vitro*	Amoebiasis	[53]
Emodin	Blocked the interaction between the spike and ACE2	*In silico, in vitro*	Cancer	[51]
Favipiravir	RNA-dependent RNA polymerase (RdRp) inhibitor	*In vitro*, clinical trial	Influenza	[49,54]
Galidesivir	Bind RdRp	*In silico*	Hepatitis C virus (HCV)	[55]
Hydroxychloroquine	Immunomodulatory effects, increase endosomal pH required for virus	*In vitro*, clinical trial	Malaria	[50,56]
Ivermectin		*In vitro*	Parasites	[57]
Lopinavir-ritonavir	inhibition of the 3CL^PRO^	*In vitro*, clinical trial	HIV	[58]
Melatonin^‡^	regulates ACE2 expression	*In silico*	Several including insomnia	[45]
Mercaptopurine	Inhibition Papain-like protease	*In silico, in vitro*	Cancer and auto-immune diseases	[45]
Remdesivir	Inhibition the RdRp/Seems to inhibit of the 3CL^PRO^	*In silico, in vitro, in vivo*	HIV/Ebola	[42,43,46,49]
Ribavirin	Inhibits RdRp	*In silico*	HCV, respiratory syncytial virus (RSV)	[59]
Saquinavir	inhibition of the 3CL^PRO^	*In silico*	HIV	[52]
Sirolimus^†^	Inhibitor of mTOR	*In silico*	Antifungal and cancer	[45]
Sofosbuvir	Bind RdRp	*In silico*	HCV	[55,59]
Tenofovir	Bind RdRp	*In silico*	HIV	[49]
Tirolone	Inducer of interferon	*In vitro*	Influenza, hepatitis, viral encephalitis and others	[60,61]
Toremifene	Destabilizing the virus membrane glycoprotein	*In silico*,* in vitro*	Cancer	[45]

† Sirolimus and dactinomycin.

‡ Melatonin and mercaptopurine.

Nanostructured Drug-Delivery Systems (NDDS) are pharmaceutical formulations capable of encapsulating, incorporating, intercalating or adsorbing different active molecules. The composition, nanoparticle size distribution, superficial charge and pH should be molecularly planned to interact with the target cells [7]. NDDS are commonly composed of different biomaterials, being processed as several pharmaceutical forms for multipurpose applications [8]. Antiviral NDDS aims to decrease drug toxicity without compromising its efficacy. It is also possible to improve the therapeutic action using lower drug concentration, due to the prolonged release profile of the entrapped drugs [9].

Different antiviral-based NDDS have been reported in the last years, mainly composed of lipids, polymers, metals and inorganic nanoparticles, as a treatment for HIV, herpes zoster, viral C hepatitis, among others viral infections [3]. Such systems based on lipids, polymers or a combination thereof deserves attention, by the low cost, biocompatibility, biodegradability and reproducibility on a large scale [10], being promisor nanocarriers to load different anti-SARS-CoV-2 drugs. In general, NDDS can be planned to target viral protease (3CLpro and PLpro), RNA polymerase (RdRp) or interact with viral S protein. Specifically, NDDS against SARS-CoV-2 can be designed to interact with viral RNA genome, surface S protein, IL 6, IFN γ-inducible protein 10 (IP-10/ CXCL10), immunoreceptor tyrosine-based activation motif (ITAM) or MCP1, in order to inhibit viral replication and infection. The nanostructured systems can also overcome biological barriers, directing the antiviral delivery to the host cells [11].

Lipid nanocarriers have shown the ability to prevent coronaviruses replication. These systems can be functionalized by active molecules-like RNase, in order to inhibit the viral replication efficiently, acting as smart delivery systems [12]. Positively charged nanocarriers, such as DOTAP-based liposomes, allows electrostatic interaction with anionic viral envelope, enhancing the antiviral delivery to the target. Phospholipids-based formulations are involved in the entry of viruses, being promisor as antiviral nanocarriers [13]. On the other hand, some biopolymers, such as chitosan and carrageenan, have been showed important intrinsic antiviral properties. Moreover, NDDS-based carbohydrate-binding agents, such as the sulfated polymers, can also change the virus entry process, blocking the viral cationic surface receptors and avoiding its interaction with heparan sulfate proteoglycan on the host cell surface [14]. However, there are no reports of NDDS as COVID-19 treatment so far. Despite these impressive advantages compared with traditional drug therapies, there is still no specific regulation of nanomaterials, which represents a delay in approval for clinical trials and uses.

This review summarized relevant information regarding general coronavirus properties linking to the main drugs candidates tested as treatments. We also proposed to apply nanotechnology as a versatile approach in the development of promising treatment against COVID-19. Therefore, we clarified the most NDDS for antiviral applications described to inspire novel developments. We have also provided a critical perspective for this current scenario, focusing in the need for multidisciplinary and governmental efforts to facilitate the development and approval of nanostructured formulations.

## Coronavirus general properties

The members of the family *Coronaviridae* have enveloped, positive-stranded RNA. Virions are spherical, 120–160 nm with a large RNA (about 27 kb long RNA that encodes nonstructural such as papain-like Protease (PLpro), RNA-dependent RNA polymerase (RdRp), main protease or chymotrypsin-like (Mpro or 3CLPRO), RNA helicase encoded by the replicase, other accessory and regulatory proteins and structural proteins (spike – S protein, envelope, membrane, nucleocapsid and others) [15]. The S protein is the primary inducer of virus-neutralizing antibodies. Besides, the S protein and hemagglutinin (HE) proteins are highly variable, which suggests extensive antigenic drift and shifts. Thus, coronaviruses can adapt to new environments or hosts through mutation and recombination, and hence they may alter host range and tissue tropism efficiently [16,17].

Coronavirus only caused mild symptoms in humans until diagnostic of SARS-CoV in 2002 [18]. MERS-CoV was another disease that affected humans with serious severity and lethality index in 2012. In 2019 SARS-CoV-2, previously called 2019-nCov was first isolated [19,20]. The disease caused by SARS-CoV-2 known as COVID-19, was considered a pandemic by the World Health Organization (WHO) and had the characteristic of being highly contagious and of rapid spread [21], which led to changes in human habits with an impacted the global economy and health.

In the subfamily *Orthocoronavirinae* there are four genera: *Alphacoronavirus, Betacoronavirus, Gammacoronavirus* and *Deltacoronavirus*. *Alphacoronavirus* and *Betacoronavirus* cause disease in humans and other mammals [22]. In domestic and livestock mammals, *Alphacoronavirus* and *Betacoronvirus* can be pathogenic [23–26].

Although those viruses belonging to the *Betacoronavirus* lineage B (SARS-CoV, SARS-CoV-2 and MERS-CoV) cause severe respiratory syndrome in humans, there are other human coronaviruses, such as: HCoV-OC43, HCovKU1, HCoV-NL63 and HCoV-229E, which may induce only mild symptoms, except for young children, elderly or immunocompetent patients, which can cause severe infections [27]. The *Betacoronavirus* lineage A (HCoV-OC43 and HCovKU1) seem to originate from rodents [28]. On the other hand, HCoV-NL63 and HCoV-229E (*Alphacoronavirus*) were possibly originated from bats [29].

Some previous studies indicated that bat species (*Rhinolophus sinicus*) probably transmitted coronavirus to other mammals, including masked-palm civet (*Paguma larvata*), raccoon dog (*Nyctereutes procyonoides*) and ferret-badger (*Melogale moschata*), from wildlife markets in China, giving rise to SARS-CoV [30,31]. Also, other reports [25,32] also showed the importance of bats as a significant reservoir and other mammals as intermediate hosts to the genetic changes of the virus, which can result in the diversification of host species and increased pathogen virulence. In COVID-19, bats were probably the reservoir of the new human pandemic virus [33]. Although epidemiology is not fully understood yet, animals as *Malayan pangolins* (*Manis javanica*) can be involved in it as well [34]. Others coronavirus strains, such as *Gammacoronavirus* is associated with avian hosts [15,22], though it has been detected in marine mammal species [35] and carnivores [36]. *Deltacoronavirus* infects birds and mammals [22,29].

## Main drugs candidates for COVID-19 treatment

Despite the several promising candidates for COVID-19 treatment, there is still no effective anti-COVID-19 drug available. Besides, most of the reports provided only data related to *in silico* and *in vitro* assays. Unfortunately, clinical trials are few and inconclusive. There are several drugs able to virus or cell inhibition, as well as immunomodulators, peptides, vitamins and antibodies, have also been studied. Table 1 summarizes the main drugs tested against MERS, SARS-CoV, SARS-CoV-2 that could be potential candidates for the treatment of COVID-19.

In general, the most candidate drugs for the treatment of SARS-CoV-2 showed high toxicity. Many protease inhibitors have side effects as dyslipidemia, insulin resistance and lipodystrophy/lipoatrophy, as well as cardiovascular and cerebrovascular effects [37–41]. Other drugs as chloroquine and hydroxychloroquine induced heart rhythm problems [42], contraindicated in patients with liver or renal impairments [43,44]. Such side effects severely affect elderly and chronic disease patients [45,46], some of the people at risk of the current pandemic.

Table 1 evidenced that despite many tested drugs as COVID-19 treatment, only a few clinical trials were performed, with no consensus regarding the effectiveness. In most cases, the drug concentration, which is effective against COVID-19 cells, is extremely cytotoxic. Low efficacy and high toxicity are the main limitations of the proposed treatments.

## Nanostructured drug-delivery systems

In 1909, Paul Ehrlich (1854–1915) described the first drug-delivery system (DDS) called ‘The Magic Bullet’. He based the study on the concept of the drug release at the specific target. This arsenic-based device consisted of a new treatment against syphilis, the most efficient anti-syphilitic agent until the penicillin discovery in 1940 [62,63]. Since then, the multidisciplinary DDS field has grown exponentially, especially those designed from nanotechnology [64].

The NDDS are based on nanometric devices with huge superficial contact area [65]. NDDS can be composed of inorganic, organic or hybrid biomaterials, allowing the encapsulation, intercalation, adsorption or incorporation of many drugs and natural compounds [8]. The major goals of NDDS are to prolong the release profile and improve the efficacy of loaded active molecules through the use of lower doses, given by the optimized interaction with the biological barriers of interest, also minimizing systemic side effects [7].

**Figure 1.**
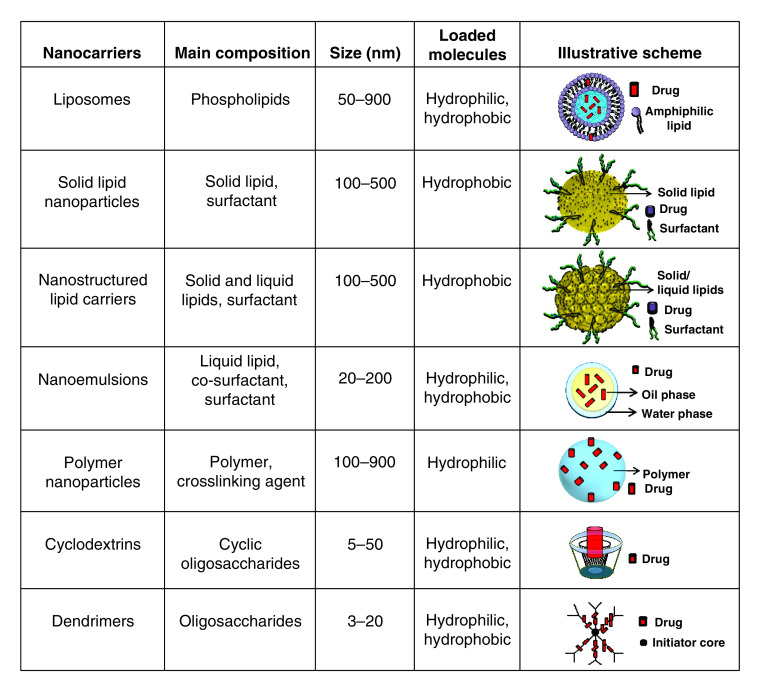
Illustrative chart of reported soft nanostructured drug delivery systems with antiviral properties, in terms of composition, nanoparticle size range (nm), ability to load molecules and its schematic representation.

Antiviral-based NDDS have been described for different applications. These systems are planned with different compositions and morphology, taking advantage of several properties, such as: its ability to penetrate the porous of biological barriers; surface functionalization to enhance its bioavailability; biomimetic structure to increase its specificity and decrease antiviral resistance [4]. Soft-based NDDS composed of lipids, as liposomes, solid lipid nanoparticles (SLN), nanostructured lipid carriers (NLC) and nanoemulsions (NE) and polymers, as nanoparticles, cyclodextrins (CD) and dendrimers (Figure 1), are NDDS with the highest antiviral activity described [3,9]. Therefore, we focused such developments in the present review. The main information of each nanocarrier abovementioned was also provided in the figure below. In each scheme, there is not a representation of its functionalization by small-molecules, linear and cyclic polymers, proteins and/or antibodies.

Among the proposed treatments, formulations against HIV are deeply noticed, as recently reviewed by Cao and Woodrow [66]. The anatomical and cellular viral reservoirs are responsible for HIV infection perpetuation in humans. Therefore, scientists have moved efforts to inhibit HIV replication. The traditional drug therapy for symptom management is successful when the patient adherence is higher than 95%; when it is lower than this, it will result in 50% treatment failures [67]. It is also necessary the administration of drugs cocktail at high concentration throughout life, severely decreasing the patient compliance to the treatments, due to the undesirable side effects [68].

In this sense, atazanavir (200 nM) is an HIV protease inhibitor that was loaded by SLN (composed of stearic acid and poloxamer 188) to enhance the antiretroviral brain delivery. The human brain microvessel endothelial cell line was employed to simulate the blood–brain barrier (BBB). SLN, with a particle size around 170 nm, allowed higher drug cellular accumulation with no description of *in vitro* toxicity, as observed for the free drug. This formulation acted as a promising treatment to prevent HIV-encephalitis and antiretroviral drug resistance [69]. In another work, immunoliposomes was described for the encapsulation of N-butyldeoxynojirimycin (NB-DNJ)-targeting cells infected with HIV. This molecule is an inhibitor of HIV gp120 folding, currently employed to prevent HIV/AIDS progression. The *in vitro* antiviral activity was carried out, and a reduction of approximately 80–95% of HIV-infected cells treated with lower doses of NB-DNJ liposomes was reported [70]. Clayton and coworkers (2009) also proposed immunoliposomes for selective delivery of HIV-1 protease inhibitor (PI1), aiming a less toxic anti-HIV therapy. PEGylated liposomes (hydrogenated soy phosphatidylcholine and cholesterol) were coated by HIV-gp120-directed monoclonal antibody F105-targeting ligand derived from HIV-gp120-directed monoclonal antibody F105. Immunoliposomes demonstrated selective uptake by HIV-1 infected cells with intracellular location, resulting in a PI1 reservoir. Moreover, higher antiviral activity was noticed for the formulation compared with free drug or conventional liposome [71].

Polymers nanocarriers have also been reported for the sustained delivery of anti-HIV drugs. Lately, CD composed of cross-linking pyromellitic dianhydride (PMDA) with two CD derivatives (methyl-β-CD-MβCD and (2-hydroxy)propyl-β-CD-HPβCD) was synthesized-loading lopinavir (protease inhibitor). The complex increased the antiviral solubility around 13-fold. The *in vitro* infectivity test demonstrated that the complex antiviral-CD allowed maximum percentage inhibition of HIV-1 cells ranging from 79 to 91%, being considered an excellent anti-HIV activity without comprise its safety [72]. Other work described poly (lactic-co-glycolic acid (PLGA) nanoparticle-loading elvitegravir to enhance viral suppression in HIV-infected macrophages. The formulation crossed the simulated BBB, with higher penetration and intracellular uptake of HIV-1-infected human monocyte-derived macrophages than free drug [73]. Moreover, biodegradable poly (lactic acid)-chitosan nanoparticles (particle size ∼300 nm) encapsulating lamivudine was provided for anti-HIV treatment for intraoral administration. Such system was not cytotoxic against mouse fibroblast cell line (L929) and prolonged the antiviral delivery for 50 h. This formulation also protected the drug degradation in the simulated gastric medium, and, consequently, the side effects [74].

Furthermore, the developments of promising treatments for other viral diseases have also been successfully provided. Donalisio and coworkers (2018) described a topical treatment against herpes zoster (HSV). A chitosan-based NE was prepared for acyclovir delivery to improve the drug permeation across the stratum corneum. NE with particles size around 200 nm showed higher ability to overcome the porcine skin barrier than free drug, assessed by *in vitro* permeation test. The enhanced anti-herpes zoster activity was noticed, with higher HSV-1 and HSV-2 infected cells uptake than control, confirmed by confocal laser scanning microscopy [75]. On the other hand, the hepatitis C virus (HCV) is an important RNA virus that affects the human's life quality. In this sense, a cationic liposome-loading apolipoprotein A-I was developed as a liver-specific siRNA targeting. The ability of the formulations to inhibit the expression of HCV proteins of mouse hepatocytes was evaluated after liposomal intravenous administration. In the first two days of treatment, a single dose of 2 mg siRNA/kg inhibited around 65–75% of viral gene expression in mouse liver, without immunotoxicity record. Additionally, the gene-silencing effect was detected for almost a week [76]. Shen and coworkers (2007) described an ophthalmic formulation for ocular HSV and cytomegalovirus (CMV) infections treatment. Liposomes (phosphatidylcholine and cholesterol)-loading ganciclovir (GCV) with encapsulation efficiency around 51% were evaluated in terms of transcorneal permeation, clearance and pharmacokinetics profile in rabbits. It was reported higher permeation capacity for liposomes than GCV solution and the clearance was similar for both samples. In pharmacokinetics, the area under the curve was almost twofold higher for liposomes than free drug, with better ocular tissue distribution and bioavailability [77].

Lately, it has been consistently demonstrated that viruses that affect the upper respiratory tract are responsible for severe symptoms and even death. Nanotechnology is an emerging tool employed to improve the traditional antiviral therapy efficacy and to provide vaccines against respiratory viruses, as recently detailed by Al-Halifa and coworkers [78]. Influenza virus infections were considered the major public health concern worldwide until a few months ago. In general, therapies were based on targeting viral proteins. However, viral variants currently lead to drug resistance. In this sense, it is opened the way for the design of pathogen-targeting antivirals, such as the vacuolar ATPase (V-ATPase) viral target, which will intercept influenza virus access in host cells [79]. PEG-PLGA nanoparticles (∼200 nm) were prepared for the sustained release of diphyllin and bafilomycin for influenza treatment, exhibiting encapsulation efficiency of 42 and 100%, respectively. The systems prolonged the release of both antiviral for 72 h, with lower cytotoxicity and higher intracellular uptake than free drugs. In a mouse sub-lethal and lethal influenza challenges, the treatment with polymer nanoparticles reduced body weight loss and viral titer in the lungs, respectively. It was also conferred a survival index 30% higher than the control [80]. Another work has proposed a treatment for influenza A virus, based on a synthesis of 6SL-PAMAM (6′-sialyllactose-polyamidoamine) dendrimers conjugates, to block viral attachment and cell entry. These dendrimer-based formulations inhibited the infection of mice lung almost tenfold more than control, preserving around 75% of mice in lethal challenge test, without induce significant weight loss (toxicity indicator) in influenza A-infected mice [81].

It is worth mentioning that the developments of antiviral-based NDDS composed of inorganic matrices also deserve attention as COVID-19 therapy. Lately, Palmieri & Pap [82] provided detailed information regarding the role of graphene as potential treatment against COVID-19. Most efforts have been directed on the development of graphene-based sensors and diagnosis tools. However, its bidimensional sheet-like structure can be functionalized by antibodies targeting viral proteins, together with the intrinsic antiviral property of graphene, being promisor as a matrix for drug/gene delivery for COVID-19 treatment [82]. On the other hand, the antiviral activity of silver nanoparticles (SN) against several viruses, such as: HIV, HSV, respiratory syncytial virus, among others, have been studied [83]. Baram-Pinto and coworkers (2009) described the synthesis of SN capped with mercaptoethanol sulfonate aiming HSV-1 treatment. The system was designed to target HSV-1 through the sulfonate groups, which interacts with viral glycoproteins, causing the blockage of *in vitro* viral permeation. SN exhibited ability to inhibit HSV-1 infection in cell culture and biocompatibility [84]. Moreover, titanium dioxide nanoparticles (TiO_2_) has also been investigated as antiviral agents, due to the ability to damage the lipid viral envelope, providing specific delivery to the viral host cell [85]. Levina and coworkers (2015) immobilized DNA fragments to TiO_2_, resulting in a nanocomposite with antiviral activity against the influenza A virus. Such system was able to deliver nucleic acids into the cells with efficiency, without the use of transfection agents [86].

Despite all advances in antiviral-based NDDS detailed above, there is still no description of a specific and effective treatment against COVID-19 so far [87]. There are only two reports of NDDS as treatments for other *Coronaviridae* family diseases. Last year, a pharmaceutical hybrid composition composed of polymer-based functionalized spermine-liposomes (100–400 nm) loading siRNA for pulmonary delivery was patented, targeting a conserved region of viral RNA. The intranasal formulation was able to release siRNA specifically in infected VERO cells, also inhibiting *in vivo* MERS-CoV gene expression in mouse [88]. Another disease caused by *Coronaviridae* family is the feline infectious peritonitis (FIP), a fatal and incurable viral infection in cats. Therefore, Hu and coworkers provided formulations composed of PEG-PGLA nanoparticles (∼40 nm) encapsulating diphyllin to act as viral V-ATPase inhibitor. The authors demonstrated that this system was biocompatible after high dose intravenously administered in 8-week old mice, decreasing FIP replication. The *in vitro* model of antibody-dependent enhancement of FIP infection showed that the polymer nanoparticles exhibited higher antiviral effect against FIP than free drug [89].

## Conclusion

The world is a testimonial of a health and global economic crisis caused by the COVID-19 pandemic. Scientists worldwide have moving efforts to understand the particularities of this viral infection and pandemic dynamics, in order to develop effective and safe treatment, which is still a challenge. Nanotechnology is a promising tool to improve the efficacy without compromise the drug safety. Different NDDS with antiviral activities were reported. It was demonstrated several advantages in comparison to traditional therapies. Finally, to cross the academic boundary, more R&D investments and specific regulation are urgent to allow NDDS can be employed as effective treatments against COVID-19.

## Future perspective

The world is going through a public health emergency which was not described since the Spanish flu (1918). There are reports of 43,147,494 cases and 1,155,553 deaths (data up to 27^th^ October 2020) of COVID-19, in at least 210 different countries and territories [21]. Scientists have running against time to understand the virus genetics and the pandemics dynamics to develop nanodevices that contribute to asepsis, prevention and treatment of patients [90].

Although prevention is always the most important action in public health, novel treatments against COVID-19 are as urgent as vaccines developments. In the last weeks, hundreds of drugs have been tested as a potential treatment for COVID-19. However, the main published works only provided *in silico* and *in vitro* results. The human coronavirus cell culture test is essential for screening of promisor candidates to perform the subsequent biological assays. Though, such models have disadvantages in understanding the interaction among tissues, cells and proteins of the target virus and biological barriers. Therefore, *in vitro* results cannot be directly correlated with clinical success in the pharmaceutical development. It should direct the most desirable candidates to *in vivo* efficacy assays. These *in vivo* achieved will determine the best candidates to be submitted to clinical trials.

The standardization of the *in vivo* SARS-CoV-2 experimental model is still a challenge. In such models, in addition to biosafety and ethics, it is not simple to reproduce this viral disease in laboratory animals [91], which can explain the delay in the publication of *in vivo* results. The use of chicken embryos is a versatile alternative to the currently employed *in vivo* models using mammals [92]. This is possible due to the viral replication of *Gammacoronavirus* IBV (infectious bronchitis virus) that causes distinguish embryonic chicken lesions. In this model, the influences of proteins and immune cells are also considered, besides the cellular interactions [93]. Furthermore, the chicken embryo model does not require ethics committee approval and is carried out with fewer animals, which are relevant advantages of this alternative *in vivo* model. Although, it would be useful only for antivirals conserved regions of coronaviruses (as functional virus proteinases) targeting or treatment with cytokines, as occurs in drugs targeting 3CL^PRO^ and RdRp polyproteins, where these proteases have conserved features in all coronaviruses [94]. The use of alternative *in vivo* models is a versatile approach to test the toxicity of NDDS in reduced time and low number of animals. *In vivo* efficacy anti-COVID-19 tests have to be standardized and immediately performed.

It is worth mentioning that bats and birds are ideal hosts for *alpha/betacoronavirus* and *gamma/deltacoronavirus*, respectively, increasing coronavirus evolution and dissemination [29]. These two hosts could be involved in the currently emerging epidemic strains, and, therefore, it should be deeply studied. In this way, the analysis of drugs that affect conserved features of the viruses should be further explored in the IBV and chicken embryos models, in attempt do find an effective treatment of the current and future pandemics.

In general, it was observed that there are only a few works that have performed *in vivo* or clinical trials, probably due to the abovementioned technical difficulties. These reports have described unsatisfactory results. In this sense, nanotechnology appears as a promising science in the development of optimized vaccines, treatment, biosensors and antiseptics against coronaviruses strains [95]. Unfortunately, we observed that the number of antiviral NDDS reports is drastically smaller than for any other drugs studied. Therefore, it is important to consider the results obtained from NDDS as treatments for other viral diseases, such as HIV, and those described specifically against other coronaviruses-based diseases, to inspire the further design of effective nanosystems against SARS-CoV-2.

Besides all these challenges, important lessons must be learned in this process. The NDDS development has to be performed with multidisciplinary efforts, with an active participation of physicists, chemists, biotechnologists, geneticists, virologists, engineers, toxicologists, pharmaceutics, veterinarians and doctors. Once all particularities of SARS-Cov-2 have been elucidated, it will be possible to design a nanodevice with specific viral targeting. Moreover, given by a prolonged release profile of entrapped drugs, it will be possible to administrate a single dose with lower drug concentration without causing systemic or local toxicity, increasing the patient compliance to the treatment.

The physicochemical and biological properties of SARS-Cov-2, antivirals and biological barriers should govern further NDDS composition, size, shape and administration route. Advanced systems will combine different nanocarriers (lipid–polymer, polymer–polymer, protein–polymer) in a single formulation, resulting in hybrid NDDS that can be processed as different pharmaceutical forms. In this approach, several advantages can be listed: preventing drug-loaded nanoparticles opsonization and macrophage phagocytose; suitable nanoparticle size, morphology and superficial electrical charge to interact/penetrate with skin, mucus, lung or BBB; combining drug sustained release with viral targeting, through inhibition of protein synthesis, gene silencing or vacuolar ATPase inhibition; improving plasma half-life and bioavailability of drugs; preventing ROS generation using antioxidant excipients; biodegradable and biocompatible properties [4,7]. Finally, it is also important to consider the possibility of development of biological treatments; use of natural compounds as drugs and better understanding of tissue damage caused by the virus. Therefore, the analyses of healed patients’ plasma and affected organs of COVID-19 victims can also provide relevant information for the design of an effective and safe COVID-19 treatment.

However, there are some drawbacks responsible for the delay in NDDS to reach the market. Once US FDA identifies that nanotechnology is a promising technology to be applied in health and food developments [96], there are some critics regarding FDA's guidance, which is still no specific for nanoparticles. The lack of regulations related to excipients purity and toxicity of degradation products are also common concerns; as well as the long-term environmental impact of nanostructured residues and scale up viability [65,97]. On the other hand, the United States National Nanotechnology Initiative/Environmental, Health and Safety Research Strategy has focused on determining physicochemical and biological parameters to ensure the efficacy and safety of nanostructured delivery systems [98]. In 2016, the International Standard Particle Tracking Analysis Guide (ISO 19430) was provided, emphasizing nanoparticle size distribution and concentration measurements as structural parameters to ensure stability of nanosystems [99]. Such entities and guides have to be considered by the FDA to allow that NDDS developments can efficiently cross the academic boundaries and contribute to people's health and life quality, as urged in this time.

We hope that this public health emergency will clarify the need for more investment in R&D, move governments and entities toward specific regulation of nanosystems and reduce bureaucracy for running clinical trials.

**Executive summary**

**Coronavirus general properties**

- The structural and genetic information of SARS-Cov-2 were provided.

**Main drugs candidates for COVID-19 treatment**

- Several drugs have been tested as a candidate to COVID-19 treatment.
- Most reports provided data related to *in silico* and *in vitro* assays.
- Clinical trials are few and inconclusive. Results are still disappointing, mainly regarding drugs toxicity.
- There is not an effective therapy against SARS-Cov-2 until now.

**Nanostructured drug-delivery systems**

- Different nanosystems have been prepared, aiming different antiviral activities.
- The nanostructured drug-delivery systems strategy to inactivate virus replication was discussed.
- *In vitro* and *in vivo* results determined biocompatibility and effectiveness of nanostructured drug delivery systems against other virus strains and coronavirus family diseases.
- Soft materials such as lipid and polymer nanoparticles have intrinsic antiviral and desirable physicochemical properties to load antivirals, a promising COVID-19 treatment.

**Future perspective**

- There are still some challenges to overcome, from the formulation design to the approval clinical uses.
- The alternative *in vivo* efficacy models should be explored and standardized, in order to facilitate the screening of the best candidates to be submitted to clinical trials.
- There are needs of regulation of nanostructured formulations, governmental investments in R&D and to reduce bureaucracy for running clinical trials.
